# Effect of Combination Treatment With Varenicline and Nicotine Patch on Smoking Cessation Among Smokers Who Drink Heavily: A Randomized Clinical Trial

**DOI:** 10.1001/jamanetworkopen.2022.0951

**Published:** 2022-03-04

**Authors:** Andrea King, Ashley Vena, Harriet de Wit, Jon E. Grant, Dingcai Cao

**Affiliations:** 1 Department of Psychiatry and Behavioral Neuroscience, University of Chicago, Chicago, Illinois; 2 Department of Ophthalmology and Visual Sciences, University of Illinois at Chicago, Chicago

## Abstract

**Importance:**

The concurrent use of both tobacco and alcohol causes substantial disease and early mortality, and smokers who drink heavily tend to be less successful in smoking cessation than smokers who do not. Although varenicline combined with nicotine replacement therapy for smoking cessation has been examined among smokers who do not drink heavily, this combination treatment has not yet been examined among smokers who drink heavily.

**Objective:**

To determine whether combined treatment with varenicline tartrate and nicotine patch improves continuous abstinence from cigarette smoking among smokers who drink heavily.

**Design, Setting, and Participants:**

This double-blind, placebo-controlled, superiority randomized clinical trial evaluated combined treatment with varenicline and nicotine patch compared with placebo and nicotine patch for smoking cessation (primary outcome) and drinking behavior (secondary outcome) among smokers who drink heavily. The clinical trial was conducted at 2 outpatient sites in Chicago, Illinois, with enrollment from March 26, 2018, to February 14, 2020. The 122 participants were recruited from the community via social media and public transit advertisements and equally randomized to the 2 treatment groups, which were stratified by sex and smoking behavior. Eligible participants smoked between 5 and 30 cigarettes per day and drank heavily (>14 drinks per week for men or >7 drinks per week for women and ≥1 heavy drinking day [defined as >5 drinks per occasion for men or >4 drinks per occasion for women] per month for the past year) and had a desire to quit smoking.

**Interventions:**

Varenicline tartrate, 1.0 mg, twice daily or matching placebo pills twice daily for 12 weeks. Nicotine patch at manufacturer-recommended doses for 10 weeks and brief individual smoking cessation counseling the week before the quit date and on the quit date.

**Main Outcomes and Measures:**

The primary outcome was self-reported continuous cigarette abstinence through weeks 9 to 12; abstinence was biochemically confirmed at the week 12 study visit. Secondary outcomes were the frequency of weekly drinking and weekly heavy drinking during the study period.

**Results:**

Among 122 participants (mean [SD] age, 44.0 [12.4] years; 67 men [54.9%]), 61 were randomly assigned to receive combined treatment with varenicline and nicotine patch (varenicline group), and 61 were randomly assigned to receive placebo and nicotine patch (placebo group). A total of 54 participants (44.3%) self-identified as Black, 56 (45.9%) as White, and 12 (9.8%) as other races (including American Indian or Alaska Native, Asian, >1 race, and unspecified race). A total of 8 participants (6.6%) self-identified as Hispanic and 114 (93.4%) as non-Hispanic ethnicity. Study retention to 12 weeks was 89%. The intention-to-treat analyses showed higher smoking cessation rates during weeks 9 to 12 in the varenicline group vs the placebo group (27 participants [44.3%] vs 17 participants [27.9%]; odds ratio, 2.20; 95% CI, 1.01-4.80; *P *= .047) and lower likelihood of relapse throughout treatment in the varenicline group relative to the placebo group (hazard ratio, 0.62; 95% CI, 0.40-0.96; *P *= .03). Both treatments were well tolerated; however, compared with participants in the placebo group, those in the varenicline group experienced more adverse effects, with 5 participants in the varenicline group discontinuing medication due to adverse effects.

**Conclusions and Relevance:**

In this study, combined treatment with varenicline and nicotine patch was more effective than placebo and nicotine patch for smoking cessation among smokers who drink heavily. The combination treatment had no effect on alcohol consumption, with both groups showing significant reductions. Combination treatment with varenicline and nicotine patch may be a viable option for smokers who drink heavily.

**Trial Registration:**

ClinicalTrials.gov Identifier: NCT02859142

## Key Points

**Question:**

Is the combination of varenicline tartrate and nicotine patch a more effective treatment than placebo with nicotine patch for smoking cessation in adult cigarette smokers who drink heavily?

**Findings:**

In this randomized clinical trial of 122 smokers who drink heavily, combination treatment with varenicline and nicotine patch significantly improved continuous cigarette abstinence during the final 4 weeks of treatment relative to placebo and nicotine patch.

**Meaning:**

This study found that combined treatment with varenicline and nicotine patch improved smoking cessation outcomes among smokers who drink heavily, a population who have historically experienced worse outcomes with standard approved treatments for tobacco cessation.

## Teaser

This randomized clinical trial assesses whether combined treatment with varenicline tartrate and nicotine patch improves continuous abstinence from cigarette smoking among smokers who drink heavily.

## Introduction

Tobacco and alcohol use are among the top 3 leading contributors to preventable disease and injury in the US and cause significant public health and economic burdens.^1,2,3^ Moreover, many people concurrently use both substances, resulting in high rates of cancer and pulmonary and cardiovascular disease, with mortality beyond the risks of each substance individually.^4,5,6,7,8^ Almost 20% of smokers drink heavily compared with 6.5% of nonsmokers; conversely, those who drink heavily are 2 to 3 times more likely to smoke than those who do not.^9^ Notably, smokers who drink heavily are less likely to quit smoking than those who drink lightly or do not drink.^10,11^ Thus, there is a need to develop efficacious interventions targeting smokers who drink heavily.

One potential candidate for targeting both alcohol and smoking behaviors is varenicline tartrate, a highly effective pharmacotherapy approved for smoking cessation.^12,13^ Varenicline may improve smoking cessation rates among smokers who drink heavily because it acts on neuronal nicotinic acetylcholine receptors that play an important role in the reinforcing effects of both alcohol and nicotine.^14,15,16^ Although varenicline is not an approved medication for alcohol use disorder (AUD),^17^ it has been shown to modify acute alcohol responses^18,19^ and reduce consumption levels.^20,21^ These effects may be important for smokers who drink heavily because they regularly drink to intoxication, which increases smoking urges^22^ and smoking lapses.^23,24^ Thus, varenicline therapy may be effective for smokers who drink heavily because of possible shared neurochemical mechanisms and because alcohol and tobacco facilitate each other.

For the treatment of smokers who drink heavily, varenicline is more effective than other approved monotherapies, such as bupropion or nicotine replacement therapy, for smoking cessation^25,26,27^ or cigarette reduction.^21^ However, even with varenicline, quit rates among smokers who drink heavily lag behind those of smokers who do not^11^; therefore, a combined pharmacotherapeutic approach may provide additional benefit. The combination of varenicline with the opioid antagonist naltrexone was recently shown to be effective in reducing the number of drinks consumed per drinking day among smokers who drink heavily but was inferior to varenicline alone for smoking abstinence.^28^ Another combination therapy approach, varenicline and nicotine patch, has been examined among smokers who do not drink heavily and was shown to be well tolerated.^29^ This combination treatment produced higher smoking cessation rates than varenicline alone,^30^ particularly among heavy smokers,^31^ but did not lead to higher quit rates when nicotine patch was delivered for a shorter interval than manufacturer recommendations.^29^ Notably, the combination of varenicline and nicotine replacement therapy has not been studied among smokers who drink heavily because they are often excluded from these types of randomized clinical trials.

Thus, we conducted a randomized clinical trial to examine the efficacy of combined treatment with varenicline and nicotine patch over 12 weeks of smoking cessation treatment among smokers who drink heavily. We hypothesized that the combined treatment would improve our primary outcome of smoking cessation rates during weeks 9 to 12 compared with placebo and nicotine patch. In addition, we hypothesized that the combined treatment would also reduce secondary alcohol drinking outcomes as measured by the frequency of weekly drinking and weekly heavy drinking throughout the 12-week treatment period.

## Methods

### Design Overview

The study was a double-blind, placebo-controlled, superiority randomized clinical trial evaluating the effect of varenicline combined with nicotine patch vs placebo combined with nicotine patch on smoking cessation (primary outcome) and weekly drinking behavior (secondary outcome) among smokers who drink heavily. The study was approved by the University of Chicago Institutional Review Board (Supplement 1). All participants provided both written and oral informed consent at the screening visit. This study followed the Consolidated Standards of Reporting Trials (CONSORT) reporting guideline for randomized clinical trials.^32^

Participants were enrolled between March 26, 2018, and February 14, 2020. Most study visits (88%) took place at the Clinical Addictions Research Laboratory at the University of Chicago, Chicago, Illinois, and the remainder (12%) took place at the Respiratory Health Association in downtown Chicago. Recruitment was conducted via advertisements on social media and public transit as well as community outreach to local organizations. Study candidates deemed eligible based on a telephone screening interview were invited to attend an in-person screening session.

Eligible candidates were age 18 to 85 years; had a current desire to quit smoking; had self-reported smoking frequency of 5 to 30 cigarettes per day, which was bioverified by urinary cotinine level (≥3 on immunochromatographic assay test strips [NicAlert; Nymox Pharmaceutical Corporation]); were fluent in English; had an educational level of grade 8 or higher; had stable residence; did not use nicotine patches within the past 3 months or receive varenicline within the past 3 years; and had no history of adverse reactions to varenicline or nicotine patch. Candidates were also required to meet hazardous drinking levels consistent with National Institute on Alcohol and Alcoholism guidelines (>14 drinks per week for men or >7 drinks per week for women and ≥1 heavy drinking day [defined as >5 drinks per occasion for men or >4 drinks per occasion for women] per month for the past year).^33^ Candidates were not seeking treatment to change their drinking behavior. Individuals with major medical or psychiatric contraindications were deemed ineligible; contraindications included uncontrolled hypertension, history of seizures, liver enzymes outside of the normal range, history of suicide attempt or current suicidal ideation, and history of severe alcohol withdrawal. Among women, current pregnancy, breastfeeding, plans to become pregnant within the next 3 months, or unwillingness to use effective birth control, as applicable, were also reasons for exclusion.

After providing informed consent at the screening visit, candidates completed surveys on demographic characteristics, medical history, and smoking, mood, and substance use history. They also completed the Fagerström Test for Cigarette Dependence (score range, 0-10 points, with higher scores indicating greater nicotine dependence),^34^ the Alcohol Use Disorders Identification Test (score range, 0-40 points, with higher scores indicating greater alcohol consumption and alcohol-related problems; potential problematic drinking was defined as a score of ≥8 points),^35^ the research version of the Structured Clinical Interview for the *Diagnostic and Statistical Manual of Mental Disorders* (Fifth Edition),^36^ the Columbia Suicide Severity Rating Scale (score range, 0-25 points, with higher scores indicating greater levels of suicidal ideation or behavior),^37^ and a Timeline Followback^38^ interview measuring daily cigarette and alcohol use within the past 28 days. At arrival, candidates were required to have a breath alcohol concentration of 0 g/dL, a score of less than 10 points on the Clinical Institute Withdrawal Assessment for Alcohol (score range, 0-67 points, with higher scores indicating greater severity of withdrawal symptoms),^39^ and a negative urine toxicologic screening result for cocaine, amphetamines, benzodiazepines, opiates, and phencyclidine. Individuals deemed eligible were scheduled for 4 study visits over 12 weeks. Randomization occurred during the first study visit, and the target smoking cessation date was scheduled for 1 week later to coincide with the second study visit. The third and fourth visits occurred at 2 weeks and 12 weeks after the target quit date, respectively.

### Randomization and Interventions

Varenicline and matching placebo tablets were provided by Pfizer. Participants were randomly assigned (1:1 via a random number generator) to receive either varenicline and nicotine patch (varenicline group) or placebo and nicotine patch (placebo group), stratified by sex and smoking behavior (light smoking [defined as <10 cigarettes per day] vs heavy smoking [defined as ≥10 cigarettes per day]) (eMethods in Supplement 2). At the first study visit, all participants in the intention-to-treat sample ingested the first tablet (varenicline or placebo) with a small snack (granola bar) under the observation of an investigator (A.V.).

The doses of varenicline tartrate were titrated according to the standard approved regimen of 0.5 mg once daily for 3 days, 0.5 mg twice daily for 4 days, and 1.0 mg twice daily starting on the quit date through the remaining 12 weeks of treatment. After week 12, participants in both groups were given the option to receive down-titration dosing. For the varenicline group, this dosing comprised 0.5-mg tablets twice daily for 4 days, then 0.5-mg tablets once daily for 3 days. Participants randomized to the placebo group received tablets that appeared identical to varenicline using the same dosing schedule. The nicotine patch was provided at the first visit, with instructions to initiate use on the morning of their quit day and to continue use for 10 weeks at dosing levels recommended by the manufacturer. Light smokers (<10 cigarettes per day) received 14-mg patches to use daily for the first 6 weeks, then 7-mg patches to use daily for 4 weeks. Heavy smokers (≥10 cigarettes per day) received 21-mg patches to use daily for the first 6 weeks followed by 14-mg patches to use daily for 2 weeks, then 7-mg patches to use daily for 2 weeks. Medication and patch adherence was monitored via tablet and patch counts at each study visit and self-reported log entries. Participants who discontinued medication or had a dose reduction were allowed to complete the trial.

Each participant received brief behavioral therapy during their first 2 visits. At the first visit, they met for 30 to 45 minutes with a trained counselor supervised by a licensed psychologist (A.K.). The counseling protocol was based on the Courage to Quit program^40^ and included preparations for quit day, discussion of medication adherence, and techniques for coping with smoking urges and nicotine withdrawal symptoms. The counseling focused on smoking cessation, and changes in alcohol drinking behavior were mentioned briefly in the motivational context of reducing smoking triggers. Participants attended a second 15-minute counseling session during the second visit (quit day) to reinforce the skills discussed in the first visit. At the fourth study visit (week 12), participants received $60 as compensation for time spent completing study measures.

### Assessments

At each visit, participants received breath tests measuring carbon monoxide and alcohol levels, measurement of vital signs and body weight, and investigator-delivered interviews, including the Clinical Institute Withdrawal Assessment for Alcohol, the Timeline Followback interview for daily cigarette and alcohol use, and the Columbia Suicide Severity Rating Scale to assure safety.^26^ Participants also completed a 15-item assessment of adverse effects (AEs) rated on a 4-point scale (with 1 indicating absent, 2 indicating mild, 3 indicating moderate, and 4 indicating severe AEs) and returned any unused tablets and patches along with a log of their daily tablet and patch use. The primary outcome was smoking cessation rates as defined by continuous cigarette abstinence based on self-report of no smoking (defined as not even 1 puff of a cigarette) during weeks 9 to 12 of treatment; smoking cessation was biochemically confirmed by an exhaled carbon monoxide level of 10 ppm or lower at the fourth study visit (week 12)^12,41,42^ (details and adjustments made due to the SARS-CoV-2 pandemic are available in eMethods in Supplement 2). Secondary outcomes were alcohol drinking measured by weekly drinking frequency and weekly heavy drinking frequency throughout the treatment period.

### Statistical Analysis

The sample size of 61 participants per group was determined based on data obtained from the largest study to date of combined treatment with varenicline and nicotine patch for smoking cessation.^30^ With this sample size, the clinical trial had at least 80% power to detect a medium effect size (Cohen *d* = 0.52) for the between-group difference on a continuous drinking outcome for a 2-sided test at α = .05. A logistic regression model was used to compare continuous smoking abstinence (not even 1 puff of a cigarette) between groups through weeks 9 to 12. A Cox proportional hazards regression model was conducted to assess continuous smoking status starting on the quit day through week 12 of active treatment. Both analyses adjusted for baseline number of cigarettes per smoking day. An intention-to-treat approach was used for analyses conducted among all randomized participants; because most participants who did not complete the clinical trial (11 of 13 individuals [84.6%]) reported continuation of smoking up to the point of study withdrawal, we determined that those who did not complete the study could be reasonably assumed to have relapsed to smoking.^43^ For changes in drinking outcomes (weekly drinking days and heavy drinking days), generalized estimating equation models (with normal distributions and identity link functions) were used to analyze the effects of group, time, and their interaction, controlling for baseline drinking behavior.

All tests were 2-sided with a significance threshold of *P* < .05. All analyses were conducted using Stata software, version 15.1 (StataCorp LLC).

## Results

### Participants

**Figure 1.**
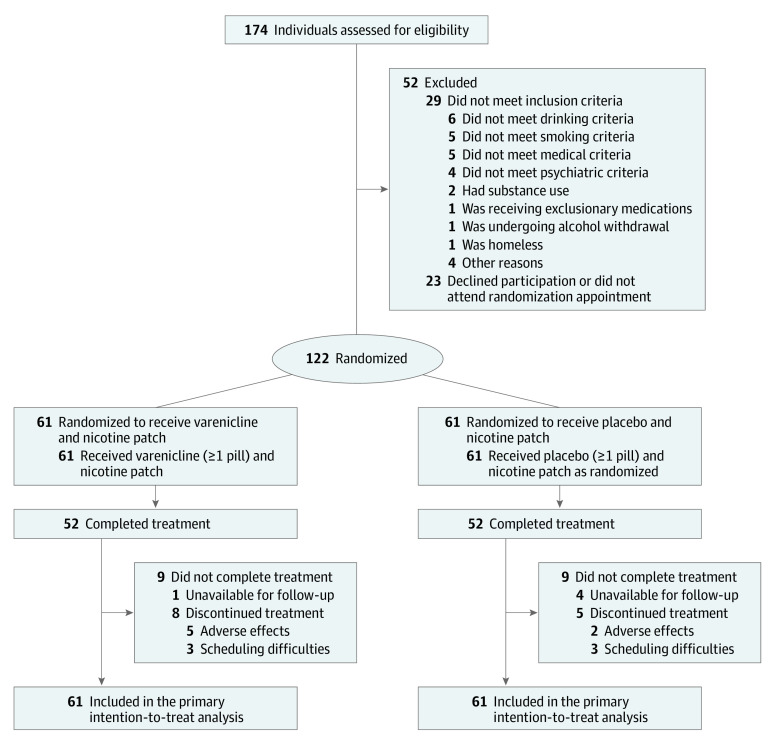
Study Flow Diagram

Among 122 participants, 61 were randomly assigned to receive combined treatment with varenicline and nicotine patch, and 61 were randomly assigned to receive placebo and nicotine patch (Figure 1). The mean (SD) age of the total sample was 44.0 (12.4) years; 55 participants (45.1%) were female, and 67 (54.9%) were male (Table 1). A total of 54 participants (44.3%) self-identified as Black, 56 (45.9%) self-identified as White, and 12 (9.8%) self-identified as other races (including American Indian or Alaska Native, Asian, >1 race, and unspecified race). A total of 8 participants (6.6%) self-identified as Hispanic and 114 (93.4%) as non-Hispanic ethnicity. The mean (SD) smoking duration was 25.2 (13.2) years, with a mean (SD) of 11.8 (6.6) cigarettes smoked per smoking day and a mean (SD) score of 3.9 (2.4) points on the Fagerström Test for Cigarette Dependence, indicating moderate dependence. All participants regularly drank heavily, with a mean (SD) score of 11.9 (5.6) points on the Alcohol Use Disorders Identification Test (scores of ≥8 points indicated potential problematic drinking). Mean (SD) alcohol consumption was 24.7 (19.6) drinks per week, with 33% constituting heavy drinking days among all days in the past month. A total of 68 participants (55.7%) met the criteria for AUD. The varenicline vs placebo groups did not differ with regard to demographic characteristics (eg, mean [SD] age, 44.0 [12.9] years vs 44.0 [12.0] years; 35 men [57.4%] vs 32 men [52.5%]), smoking patterns and use (eg, mean [SD] smoking days in past month, 27.8 [0.8] vs 27.4 [1.7]; mean [SD] cigarettes per smoking day, 11.8 [6.3] vs 11.8 [7.1]), and drinking patterns and use (eg, mean [SD] drinking days per week, 4.4 [1.8] vs 4.7 [1.8]; 30% vs 37% heavy drinking days).

**Table 1.** Participant Characteristics

Characteristic	No. (%)		
	Total (N = 122)	Varenicline and nicotine patch (n = 61)	Placebo and nicotine patch (n = 61)
Age, mean (SD), y	44.0 (12.4)	44.0 (12.9)	44.0 (12.0)
Sex			
Female	55 (45.1)	26 (42.6)	29 (47.5)
Male	67 (54.9)	35 (57.4)	32 (52.5)
Race			
Black	54 (44.3)	24 (39.3)	30 (49.2)
White	56 (45.9)	28 (45.9)	28 (45.9)
Other^a^	12 (9.8)	9 (14.8)	3 (4.9)
Ethnicity			
Hispanic	8 (6.6)	6 (9.8)	2 (3.3)
Non-Hispanic	114 (93.4)	55 (90.2)	59 (96.7)
Educational level, mean (SD), y	14.7 (2.3)	14.5 (2.3)	14.8 (2.3)
BDI score, mean (SD)^b^	5.9 (6.1)	5.8 (4.9)	6.1 (7.1)
Smoking patterns and use			
Duration of smoking, mean (SD), y	25.2 (13.2)	25.5 (13.9)	24.9 (12.6)
Smoking days in past month, mean (SD)	27.6 (1.3)	27.8 (0.8)	27.4 (1.7)
Cigarettes per smoking day, mean (SD)	11.8 (6.6)	11.8 (6.3)	11.8 (7.1)
Menthol cigarette preference	67 (54.9)	32 (52.5)	35 (57.4)
FTCD score, mean (SD)^c^	3.9 (2.4)	4.2 (2.4)	3.6 (2.4)
Carbon monoxide level, mean (SD), ppm	17.1 (10.5)	16.7 (11.3)	17.5 (9.7)
Drinking patterns and use			
Drinking days per week, mean (SD)^d^	4.5 (1.8)	4.4 (1.8)	4.7 (1.8)
Drinks per drinking day, mean (SD)^d^	5.7 (4.0)	5.4 (3.7)	6.1 (4.2)
Heavy drinking days within past month, %^d^	33	30	37
AUDIT total score, mean (SD)^e^	11.9 (5.6)	11.3 (5.1)	12.6 (6.1)
No. of *DSM-5* AUD symptoms			
Mean (SD)^f^	2.5 (2.2)	2.6 (2.0)	2.5 (2.5)
Symptom category			
None (0-1)	52 (42.6)	24 (39.3)	28 (45.9)
Mild (2-3)	34 (27.9)	17 (27.9)	17 (27.9)
Moderate (4-5)	23 (18.9)	14 (23.0)	9 (14.8)
Severe (≥6)	13 (10.7)	6 (9.8)	7 (11.5)

Abbreviations: AUD, alcohol use disorder; AUDIT, Alcohol Use Disorders Identification Test; BDI, Beck Depression Inventory; *DSM-5*, *Diagnostic and Statistical Manual of Mental Disorders* (Fifth Edition); FTCD, Fagerström Test for Cigarette Dependence.

^a^ Other category included American Indian or Alaska Native, Asian, more than 1 race, and unspecified race.

^b^ Score range, 0-63 points, with higher scores indicating more severe levels of depression.

^c^ Score range, 0-10 points, with higher scores indicating greater nicotine dependence.

^d^ Consumption during the 28 days before enrollment.

^e^ Score range, 0-40 points, with higher scores indicating greater alcohol consumption and alcohol-related problems. Heavy drinking days were defined as more than 5 drinks per occasion for men and more than 4 drinks per occasion for women.

^f^ Missing data for 2 participants (1 participant from each group) were imputed based on AUDIT scores and drinking patterns.

### Retention and Adherence

Study retention through 12 weeks of treatment was high, with 109 of 122 participants (89.3%) completing active treatment (4 participants in the varenicline group and 9 participants in the placebo group withdrew from the study). The treatment groups did not differ in the percentage of dispensed tablets reported taken over the 12-week treatment period (median, 85.5% [IQR, 48.6%-97.2%] in the varenicline group vs 86.6% [IQR, 69.8%-97.5%] in the placebo group; *P* = .36) or the percentage of dispensed nicotine patches used (median, 82.9% [IQR, 45.7%-98.6%] in the varenicline group vs 92.9% [IQR, 67.9%-97.9%] in the placebo group; *P* = .30).

### Outcomes at End of Treatment

**Figure 2.**
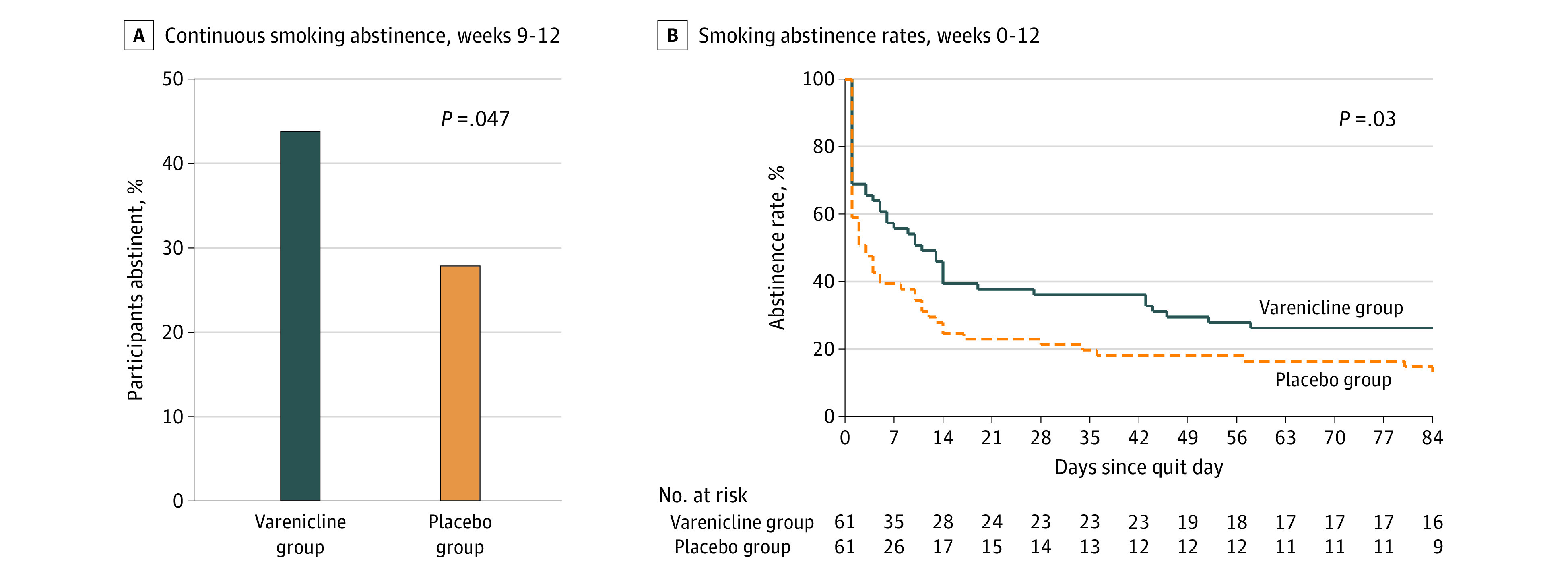
Cigarette Smoking Outcomes by Treatment Group Participants in the varenicline group received varenicline tartrate, 1.0 mg, twice daily and nicotine patch. Participants in the placebo group received matching placebo pills twice daily and nicotine patch. A total of 61 participants were in each group. B, Day 0 indicates the smoking cessation day, which corresponded to the first day of the full medication dose and nicotine patch.

Smoking cessation rates during weeks 9 to 12 were higher in the varenicline group vs the placebo group (27 participants [44.3%] vs 17 participants [27.9%]; odds ratio, 2.20; 95% CI, 1.01-4.80; *P* = .047) (Figure 2A). A Kaplan-Meier survival analysis of continuous cigarette abstinence throughout the 12 weeks of treatment confirmed a lower likelihood of smoking relapse among those in the varenicline group compared with those in the placebo group (hazard ratio, 0.62; 95% CI, 0.40-0.96; *P* = .03) (Figure 2B).

**Figure 3.**
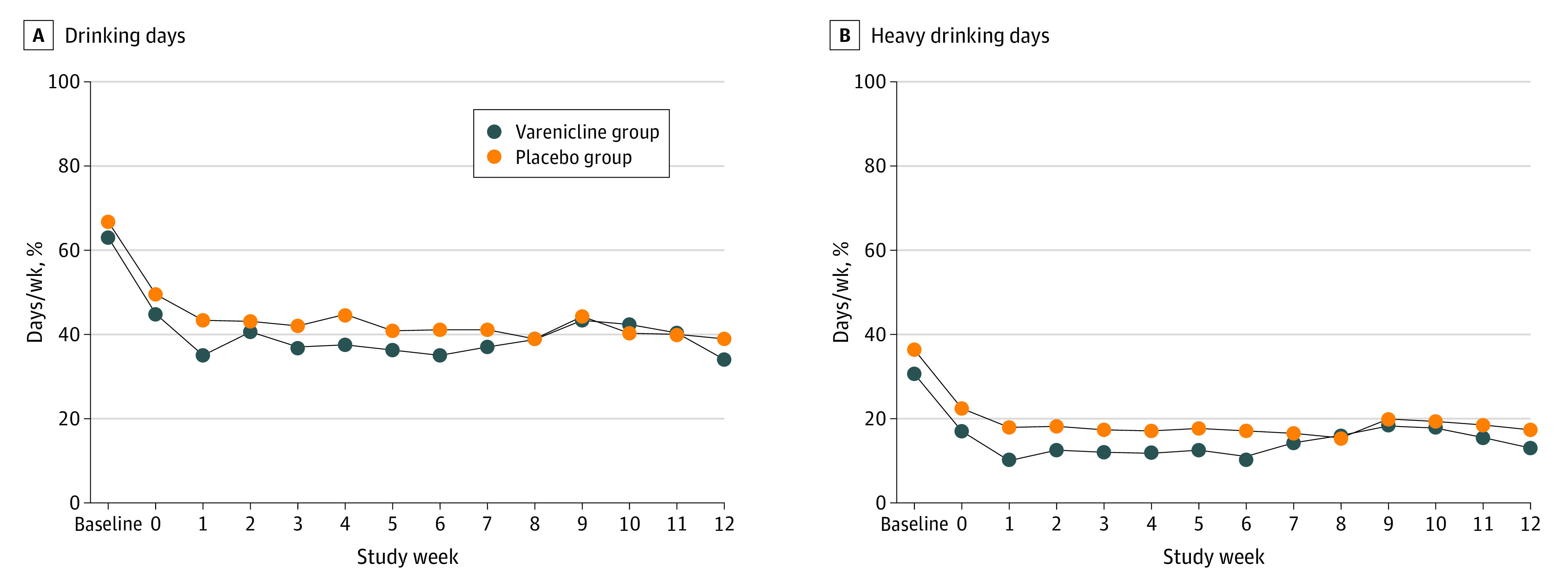
Alcohol Drinking Outcomes by Treatment Group Participants in the varenicline group received varenicline tartrate, 1.0 mg, twice daily and nicotine patch. Participants in the placebo group received matching placebo pills twice daily and nicotine patch. Analysis included 109 of 122 participants (89.3%) who completed active treatment. Baseline refers to the mean of the 4-week period before enrollment, and week 0 was the week before the smoking cessation date during which medication was initiated. For baseline drinks per week, data from 3 participants (with outlier values >3 SD higher than the mean) were normalized to 3 SD of the sample mean before analysis. Generalized estimating equation analyses were based on mean drinking days per week and heavy drinking days per week. Each week was divided by a constant of 7 for ease of presenting percentages of drinking days and heavy drinking days. Heavy drinking days were defined as 5 or more drinks per occasion for men and 4 or more drinks per occasion for women.

Regarding secondary outcomes, significant decreases in the frequency of any alcohol drinking (Figure 3A) and heavy drinking (Figure 3B) were observed. Among both groups, weekly drinking days decreased by 25% (from 65% at baseline to 49% at 12 weeks; *P* = .003 for main effect of time), and weekly heavy drinking days decreased by 54% (from 33% at baseline to 15% at 12 weeks; *P* = .004 for main effect of time). However, the treatment groups did not differ for either outcome (drinking days: χ^2^_1_  = 0.06 [*P* = .81 for main effect] and χ^2^_12_ = 10.41 [*P* = .58 for interaction]) between group and time; heavy drinking days: *P* = .57 for main effect of group and χ^2^_1_  = 0.32 [*P* = .57 for main effect] and χ^2^_12_ = 8.40 (*P* = .75 for interaction]).

### Adverse Events

The rate of AEs among participants who reported symptoms of greater severity compared with baseline are shown in Table 2. Participants in the varenicline group vs the placebo group experienced significantly more frequent nausea (34 participants [55.7%] vs 18 participants [29.5%]; *P* = .003), gas (30 participants [49.2%] vs 17 participants [27.9%]; *P* = .009), abnormal dreams (45 participants [73.8%] vs 30 participants [49.2%]; *P* = .007), sleep problems (31 participants [50.8%] vs 15 participants [24.6%]; *P* = .003), and headaches (26 participants [42.6%] vs 14 participants [23.0%]; *P* = .02) (Table 2). Among both groups, most of these AEs (190 of 220 reports [86.4%]) were rated as mild or moderate. Two serious AEs occurred in the varenicline group; 1 participant had a brief psychiatric hospitalization for suicidal ideation, and another participant had a brief hospitalization for temporary loss of consciousness. In both cases, varenicline was promptly discontinued, and participants completed the clinical trial. In the varenicline group, 5 participants (8.2%) discontinued medication because of AEs, and another 6 participants (9.8%) requested a 50% dose reduction. There were no serious AEs, discontinued medications, or dose reductions requested among participants in the placebo group.

**Table 2.** Adverse Events

Event^a^	Participants, No. (%)		*P* value^b^
	Varenicline and nicotine patch (n = 61)	Placebo and nicotine patch (n = 61)	
Nausea	34 (55.7)	18 (29.5)	.003
Vomiting	9 (14.8)	3 (4.9)	.07
Gas	30 (49.2)	17 (27.9)	.009
Constipation	22 (36.1)	12 (19.7)	.05
Abnormal dreams	45 (73.8)	30 (49.2)	.007
Sleep problems	31 (50.8)	15 (24.6)	.003
Increased heart rate	10 (16.4)	6 (9.8)	.27
Seizures	0	0	>.99
Increased effects of alcohol (drunkenness)	17 (27.9)	12 (19.7)	.29
Suicidal thoughts	2 (3.3)	1 (1.6)	.56
Dizziness	12 (19.7)	12 (19.7)	>.99
Headache	26 (42.6)	14 (23.0)	.02
Joint pain	12 (19.7)	14 (23.0)	.62
Skin irritation (outside of patch site)	12 (19.7)	9 (14.8)	.49
Other	5 (8.2)	4 (6.6)	.73

^a^ Adverse events reported as new onset or greater severity than baseline at visits 2, 3, and 4.

^b^ Significance threshold was χ^2^ *P* < .05.

## Discussion

In this randomized clinical trial, combined treatment with varenicline and nicotine patch was more effective than nicotine patch alone for smoking cessation rates during weeks 9 to 12 of treatment among smokers who drank heavily. The combined treatment had no effect on alcohol consumption. Notably, the findings of this study expanded our knowledge of smoking cessation treatment among smokers who drink heavily in several ways. First, the combination treatment of varenicline and nicotine patch improved on the historically worse smoking cessation outcomes among smokers who drink heavily, with a 44.3% quit rate that was comparable with quit rates (22%-55%) reported among smokers who did not drink heavily receiving the same combination treatment in other studies.^29,30,31,44^

Second, the results demonstrated that smokers who drink heavily can be enrolled and retained in a smoking cessation treatment program and tolerate a comprehensive pharmacotherapy regimen. Third, varenicline and nicotine patch reduced the likelihood of relapse throughout treatment, which suggests that longer-term dosing with varenicline would be necessary for its therapeutic effect to be observed.^12,13^

Although participants were enrolled irrespective of desire to change alcohol use, we observed a significant decrease in drinking frequency during treatment. This finding was unexpected because drinking modification was not a treatment goal and was not addressed beyond a brief mention of triggers during the first counseling session. Nevertheless, these participants reduced their drinking to a level similar to that observed in a study of individuals with AUD who were motivated to change their drinking behavior.^45^ This reduction in drinking may have produced a floor effect, obscuring any potential benefit of varenicline therapy for alcohol use. However, the change in drinking behavior occurred in both the varenicline and placebo groups, raising the possibility that the nicotine patch was sufficient to decrease drinking because it was able to reduce subjective intoxication and latency to drink^46^ as well as alcohol withdrawal symptoms.^47^ Participants may alternately have changed drinking behavior due to assessment reactivity, demand characteristics, or concerns about medication interactions. Warnings about varenicline from the US Food and Drug Administration suggest that patients “reduce the amount of alcohol they consume until they know whether [the medication] affects them.”^48^^(p1)^ Nonetheless, the lack of a direct varenicline benefit for drinking behavior was consistent with results from other studies involving both smokers who drink heavily^11,20,26^ and individuals with AUD.^17,45,49,50^

The combination of varenicline plus nicotine replacement therapy has been examined in clinical trials of smokers who do not drink heavily and was shown to be well tolerated but with mixed efficacy.^29,30,31,44^ The precise neuropharmacological mechanism underlying the efficacy of varenicline and nicotine patch is unclear but may be due to synergistic mechanisms of action at neuronal nicotinic acetylcholine receptors. Varenicline is more potent than nicotine patch in desensitizing reward-mediating α4β2 neuronal nicotinic acetylcholine receptors, blocking nicotine receptor occupancy,^51^ and producing synaptic trapping,^52^ but varenicline may not fully saturate α4β2 subunit receptors or provide complete blockage of nicotine reinforcement.^53^ Nicotine patch produces greater and more immediate activation of α4β2 subunit receptors and may complement varenicline by reducing ad hoc cravings that are especially important early in a smoking cessation attempt.^51^ More therapeutic clinical trials and basic translational research are needed to fully understand the interrelationship of neuronal nicotinic acetylcholine receptors and other circuitries underlying nicotine-alcohol interactions^54^ because combination treatments do not always show benefit; for example, varenicline and naltrexone were found to worsen smoking cessation rates among smokers who drink heavily compared with varenicline and placebo.^28^

### Strengths and Limitations

This study has strengths. These strengths include enrollment of a racially diverse sample of smokers who drank heavily as well as high retention rates (89% completion at 12 weeks) within a protocol that was consistent with the clinical practice of 2 visits after the smoking cessation date over 12 weeks.

The study also has limitations. First, the clinical trial met its recruitment goal, but the sample was small, precluding analyses of moderators of varenicline effects, such as sex^26^ and race.^42^ Second, this superiority clinical trial with 2-group randomization lacked the full 2 × 2 design with placebo conditions for nicotine patch; therefore, assessment of varenicline alone or double placebo–condition effects was not possible. Third, the clinical trial was completed during the early stages of the COVID-19 pandemic, and biochemical verification of self-reported smoking cessation rates was not possible for some participants given the unavoidable limitations and quick transition to completing the study using virtual and telephone platforms.^55^

## Conclusions

The findings of this randomized clinical trial indicate that, among smokers who drink heavily, more than 50% of whom had AUD, the combination of varenicline and nicotine patch treatment (vs placebo and nicotine patch) produced higher smoking cessation rates throughout 12 weeks of treatment. The combination pharmacotherapy was also safe and well tolerated. The results support continued investigation of varenicline with nicotine patch as a combination treatment strategy for smokers with hazardous drinking behaviors who have historically experienced worse outcomes with standard approved tobacco cessation treatments. Smokers who drink heavily can be enrolled and retained in smoking cessation clinical trials, and further investigation is warranted to help reduce the health disparities associated with chronic smoking and heavy drinking.

## Notes

Supplement 1.
Trial Protocol and Statistical Analysis Plan

Supplement 2. **eMethods. **Randomization, Blinding, and Bioverification of Self-reported Smoking Status
eReferences

Supplement 3.
Data Sharing Statement
