# Supplementary material for: Effect of Combination Treatment With Varenicline and Nicotine Patch on Smoking Cessation Among Smokers Who Drink Heavily: A Randomized Clinical Trial
Source: JAMA Netw Open. 2022 Mar 4;5(3):e220951. doi: 10.1001/jamanetworkopen.2022.0951 (PMC8897753; doi:10.1001/jamanetworkopen.2022.0951)
Supplement: Supplement 1. — Trial Protocol and Statistical Analysis Plan [file jamanetwopen-e220951-s001.pdf]

Supplemental Trial Protocol, Statistical Analysis Plan, and Summary of Protocol Changes for

Effect of Combination Treatment with Varenicline and Nicotine Patch on Smoking Cessation Among Heavy Drinking Smokers: A Randomized Clinical Trial

I. **Original Protocol at Trial Initiation,** page 1
II. **Summary of Changes to the Protocol,** page 14

I. <u>Original Protocol at Trial Initiation</u>

Title of Research Project: Varenicline Augmentation of Patch Outcomes in Heavy Drinkers' Smoking Cessation
Principal Investigator: Andrea King, Ph.D.
Date: 12/1/2015
Targeted Enrollment: The targeted enrollment for this protocol is 120 heavy drinking smokers.

Detailed Protocol Narrative:

1. Background

Tobacco and alcohol use are two of the top three leading causes of preventable disease worldwide, contributing to nearly seven million deaths each year[1-3]. Co-use of these substances is widely prevalent[4-8] and associated with numerous medical problems including cancers, pulmonary, and cardiovascular disease with synergistic mortality increases from esophageal, laryngeal, and oral cancers beyond the risks of each substance individually[9-11]. No treatments have been targeted to improve quit rates for heavy drinking smokers (HDS) but such treatments may be most effective if they affect both smoking and drinking behaviors and underlying aspects of inhibitory control and impulsivity[12-14]. Thus, there is a crucial need to understand the mechanisms of action of promising treatments in order to reduce the health burden of alcohol-smoking comorbidity. Our increasing knowledge about the neurocircuitry and behavioral pharmacology of alcohol and nicotine suggest that the neuronal nicotinic acetylcholine receptors (nAChRs) may be strong candidate targets in HDS to augment nicotine agonist treatment which reduces tobacco withdrawal producing modest but meaningful improvements in quit rates versus "cold turkey" unassisted quit attempts.

VAR tartrate (VAR) is a partial agonist at nAChRs that is particularly selective for the α2β4 subunit subtype thought to mediate withdrawal and smoking reward[15-17]. VAR is approved for nicotine dependence in general adult smokers with quit rates at 12 weeks at 44% vs. 18% for placebo, reduced to 23% and 10%, respectively at 52 weeks[18]. Quit rates are higher when VAR is combined with nicotine patch than with VAR alone (odds ratio, 2.13 at 6 months)[19]. The mechanism of action may involve partial nicotine agonist effects to relieve negative affect and smoking urge[15,20, 51] as well as improve working memory[21,22] and inhibitory control[23.]

We have shown that VAR improves alcohol's impairment of visual processing[24] without adverse effects on cardiovascular function[25,26]. VAR may also reduce urge to smoke[27-30] with quit rates increasing over the first few weeks of treatment unlike other medications that show declining quit rates over time[18]. HDS have been largely excluded in these aforementioned studies[31], leaving health care providers unsure of clinical approaches other than relying on the standard protocol of patch and counselling. However, this approach is less effective in HDS, as our research shows 14% quit rates in HDS compared with 24-32% in light and non-drinkers, respectively[32]. Another pharmacotherapy, naltrexone, augmented patch and counselling outcomes in HDS to 32% quit rates, higher than the 10-25% observed in non- and light-drinkers. Naltrexone is not approved for smoking cessation and so its use may be limited only to an off-

label purpose. However, VAR is approved for smoking cessation and given the complexity of smoking-alcohol interactions, VAR may be a more amenable choice for providers to augment standard treatment of patch and counselling in HDS. The rationale behind this is that VAR may increase negative-like alcohol responses and decrease drinking behaviors[24, 33-36] and its effects on alcohol sedation and intoxication are now included as a warning label. Concerns about VAR side effects and such warnings have unduly limited its use in some contexts, and in particular, for HDS. Unfortunately, data are lacking in a real-world context on the effectiveness of a comprehensive strategy to address smoking quit rates and decrease alcohol behaviours, even in patients for whom the goal of reducing or quitting drinking is not explicitly stated.

Thus, we propose to provide real-world effectiveness data on VAR as an augmentation strategy for patch and counselling. We feel that this comprehensive strategy of VAR and patch may be most helpful in HDS as they are historically difficult to treat and may be more amenable to VAR in the context of augmenting patch and brief counselling effects, as quit rates are higher with this combination than with VAR alone.[19]

2. Purpose or Hypothesis

In the proposed research, we will conduct a real-world clinic-based smoking cessation study to examine the augmentation strategy of VAR and patch versus standard treatment of patch only in HDS. VAR is approved for smoking cessation, but not routinely given in practice for HDS patients in general due to concerns about recent warnings about drinking, particularly in patients who are not yet ready to abstain from drinking. Providers are reluctant to prescribe bupropion due to seizure threshold concerns, thereby leaving patch and other forms of NRT as the main medication options. Patch is the easiest and most widely accepted of NRT strategies but data in HDS show that they are not as responsive to this standard treatment as their light and non-drinking smoker counterparts.

In this proposal, all participants will receive brief clinic counselling designed to bolster and motivate use of online self-help materials. The only exclusion criteria will be major uncontrolled medical or psychiatric conditions for which use of the nicotine patch or VAR are contraindicated. We will ascertain smoking urge and withdrawal, negative affect, neurocognition, and alcohol and smoking behaviors at pre-quit baseline, 2 and 12 weeks post quit date, and again at 26-week follow-up, i.e., 12 weeks after medication discontinuation.

Our goal is to examine:

1). Whether VAR improve smoking and drinking outcomes in HDS.
***Primary prediction**:* VAR will improve quit rates relative to patch treatment.
Secondary prediction: VAR will reduce alcohol drinking behaviours (drinks per week, % heavy drinking days) relative to patch treatment.

2). Whether VAR will decrease smoking urge, tobacco withdrawal, negative affect, and neurocognition in HDS.
***Secondary Predictions**:* VAR will improve tobacco urge, withdrawal symptoms, affect, and cognition relative to patch treatment.

3). (Exploratory aim) Whether there are individual differences that predict better treatment response with VAR. Participant characteristics and behavioural factors (age, sex, medication adherence, affect, urge, alcohol, and smoking) will be examined in multivariate analyses to identify predictors of treatment response (quit rates, drinking).

Overall impact: This investigation has the potential to identify a potentially viable option for smoking cessation in HDS that can be implemented in practice. The study will also determine who is most likely to benefit from VAR in this real-world context.

3. Protocol Methodology

Participants will be enrolled on a continuous basis. There will be 4 total in-person study visits over the trial (pre-quit, quit date, week 2 and week 12), aligned with clinical practice, ending 12 weeks after the quit date. Biochemical verification from breath tests for CO, as well as vital signs and weight, will be measured at each visit along with survey responses. These will also be used at a 26-week follow-up by telephone with biochemical verification for CO in those reporting being smoke-free.

Screening and Randomization

Participants will respond to advertisements and will undergo a brief phone screening to determine initial eligibility requirements. Qualified candidates will be invited into the lab to conduct a short screening and study information session at the Clinical Addictions Research Laboratory at the University of Chicago. At screening, participants will sign an informed consent document. Next, demographics, smoking, alcohol and substance use patterns, health history, medications, vital signs, a urine test (for pregnancy and/or drug toxicology), and a blood test will be obtained by the study nurse. Specifically, we will administer the Timeline Followback (TLFB)[39] for past month smoking and drinking, the Alcohol Dependence Scale[40] the Fagerstrom Test of Nicotine Dependence[41], the Smoking Stages Ladder Questionnaire to discern desire to quit smoking, and the Shipley Institute of Living Scale[42] (for estimated verbal ability and IQ).

Eligible participants will be randomized into one of two treatment groups: Standard Treatment (n=60) will proceed with the study receiving nicotine patches and brief counseling sessions; Augmented Treatment (n=60) will proceed with the same nicotine patches and brief counseling sessions, but will also receive standard dosing of Varenicline tartrate (VAR).

Nicotine Patches

Nicotine patches will be utilized starting at study quit date (Study Week 0), and proceed according to package insert directions (10+ cigs/day smokers will begin with 21mg patches for six weeks, followed by 14mg patches for four weeks, and finally 7mg patches for two weeks. Those smoking fewer than 10 cigarettes/day will follow the same process starting at the 14mg patch level.

Varenicline Tartrate

Those in the VAR group will receive varenicline in this effectiveness study. They will undergo an up-titration week prior to the quit date, 12 weeks of target dosing, and a down-titration week. As per Pfizer recommendations, up-titration will be 0.5mg tablets once daily for 3 days followed by twice daily for four days leading to the quit date on day 8. We will reverse this sequence for a down-titration week on week 13, which we have found in prior studies helps with patient expectancy and concerns about abrupt medication discontinuation, therefore, 22 doses of 0.5 mg per participant. Target dosing will be at the approved dose of 1mg twice daily for 12 weeks, therefore, 168 doses of 1.0 mg per participant.

Study Visits and Brief Counseling Sessions

Participants will have the choice to complete study visits at either the Clinical Addictions Research Laboratory (CARL) at the University of Chicago or at the Respiratory Health Association (RHA) in the Chicago Loop area.

Smoking Cessation Behavioral Sessions: Participants will attend one-on-one behavioral counseling sessions with a trained Masters or PhD. Level therapist at each study visit. Behavioral sessions will

involve teaching behavioral skills to assist with smoking cessation, preventing relapse, and coping with physical or emotional changes associated with cravings.

Subjective Measures: At each visit, brief self-report surveys (~10 minutes) will be given, as often used in routine practice in the smoking clinic for safety checks and monitoring of progress. These include the TLFB for daily estimates of cigarettes, alcohol drinks, as well as other forms of tobacco, cannabis or electronic cigarettes, if appropriate, because use of these substances and devices have been increasing and may affect treatment outcomes); the 10-item B-QSU[43] for smoking urge assessment; the 8-item Alcohol Urge Questionnaire[44]; the 8-item Minnesota Nicotine Withdrawal Scale[45], the Beck Depression Inventory for current depressive symptoms, and the State-Trait Anxiety Inventory to measure current anxiety. For participants who reported having smoked, a cigarette evaluation scale will be given to assess reward, pleasure, and other sensations of smoking. Safety checks will include the Clinical Institute Withdrawal Assessment (CIWA-Ar)[46], the Columbia Suicide Severity Rating Scale, and the adverse effects checklist consistent with the symptoms reported in the Physician's Desk Reference for VAR and nicotine patch.

Objective: At each visit, noninvasive objective measures will be conducted. These include carbon monoxide (CO) expired air breath tests (Smokerlyzer®) to verify nonsmoking self-report; breath alcohol content (Alco-Sensor III), vital signs (Dinamap ® ProCare Monitor, Waukesha, WI), and weight (Tanita digital scale).

Neurocognitive: At baseline and again at the 2- and 12-week study visits, participants will complete a computerized neurocognitive test battery (~15 minutes) assessing select domains of executive functioning implicated previously in the pathogenesis and maintenance of addiction. Cognitive flexibility will be measured using the Wisconsin Card Sorting Task[47]; response inhibition will be measured using the Stop Signal Task[48]; and working memory capacity will be assessed using the N-Back task[49]. Task order will be counterbalanced across groups.

Follow-Up Interview (Week 26)

At Study Week 26, participants will complete a follow-up telephone interview, completing similar subjective measures as those completed during study visits. Participants reporting being smoke-free during this interview will arrange for biochemical verification of this status via expired CO testing either by arranging for a time to stop into one of the study sites or by arranging for study staff to meet with them in their home or workplace.

Study Measures and Tasks

**Measure/Task Name**	**Screening/ Study Info Session**	**Study Visits**				**Follow-Up**
		Pre-Quit Week -1	Quit Date Week 0	Study Week 2	Study Week 12	Study Week 26
Demographics Questionnaire	**X**					
Substance Use Questionnaire	**X**					
Health History Questionnaire	**X**					
Alcohol Dependence Scale[40]	**X**					
Fagerstrom Test of Nicotine Dependence[41]	**X**					
Shipley Institute of Living Scale[42]	**X**					
Smoking Stages Ladder	**X**					

Questionnaire						
DSM-5/ICD-10	X					
Blood Test (Hepatic Panel)	X					
Physical Exam	X					
Vital Signs	X	X	X	X	X	
Weight	X	X	X	X	X	
Carbon Monoxide (CO)	X	X	X	X	X	X
Breath Alcohol Content (BrAC)	X	X	X	X	X	
Urine Toxicology and Pregnancy Testing	X					
TLFB[39]	X	X	X	X	X	X
Beck Depression Inventory	X	X	X	X	X	X
State-Trait Anxiety Inventory	X	X	X	X	X	X
BQSU[43]		X	X	X	X	X
Alcohol Urge Questionnaire (AUQ)[44]		X	X	X	X	X
Minnesota Nicotine Withdrawal Scale[45]		X	X	X	X	X
Clinical Institute Withdrawal Assessment (CIWA-AR)		X	X	X	X	X
Columbia Suicide Severity Rating Scale		X	X	X	X	X
Adverse Events Checklist		X	X	X	X	X
Wisconsin Card Sorting Task[47]		X		X	X	
Stop Signal Task[48]		X		X	X	
N-Back Task[49]		X		X	X	

4. Probable Duration of Protocol

Study activities are expected to last approximately 1 year from protocol initiation, enrolling approximately 10-15 participants per month into the trial.

5. Location of Research

At the University of Chicago, study activities will be conducted within the Clinical Addictions Research Laboratory (Billings Hospital, Rm M360). Additional visits will have the option of being conducted at the Respiratory Health Association (RHA; 1440 W Washington Blvd, Chicago, IL 60607). Both facilities are within 8 miles in Chicago IL and have clinic stop-smoking programs in place. They have partnered before for smoking cessation trials and can facilitate the proposed study by conducting effectiveness work within their smoking cessation clients to screen for current alcohol drinkers and meet enrollment estimates of 120 completed participants in a one-year study. They are complimentary service-oriented programs with approximately 500-800 square feet of available space and several offices and participant interview rooms.

6. Special Precautions

When signing informed consent, subjects will be informed that they can withdraw at any time from the study, for whatever reason, without prejudice. All data specifically obtained for research will be kept strictly confidential. Raw self-report data will be kept in a locked file cabinet, while data stored in the password protected computer database will identify subjects only by subject number and not by individual name. Access to identifying data will be limited to research staff engaged in the project. As part of the consent procedure, subjects will be advised of precautions taken to preserve confidentiality.

There will be three levels of protection for participants against the risk of medication and patches:

a) Screening and Consent: As detailed above, participants will be carefully screened via history and laboratory testing in order to eliminate those persons with elevated risks of taking nicotine patch or varenicline. This screen will exclude persons with a history of past adverse reactions to drug, pregnancy, lactation, current substance dependence, history of hepatic dysfunction or active cardiovascular disease, or medical conditions associated with an increased risk of adverse events.

b) Monitoring: During active medication treatment, participants will be monitored for adverse reactions to medications. Participants will be advised to report adverse reactions immediately and given two 24-hour contact lists:
   - For emergencies: the Study Physician and the PI phone numbers and pagers
   - For general information: the Project Coordinator's phone numbers

c) Adverse Event Reporting: Subjects will complete side effects checklists. Participants reporting side effects will be evaluated by the study physician. The study physician may order appropriate laboratory tests to follow up on side effects or adverse events.

7. Description of Experimental Controls and Use of Placebos

The protocol will be an effectiveness trial and, thus, will be addressing the effect of adding varenicline to standard patch treatment in heavy drinking smokers under "real world" clinical settings. Main dependent variables will be compared between those in the standard treatment and augmented treatment to discern success.

8. Type and Number of Experimental Subjects

N=120 heavy drinking smokers (HDS) randomized into one of two treatment conditions: 1) Standard treatment of nicotine patches and brief counseling sessions [n=60]; or 2) Augmented treatment of nicotine patch, brief counseling sessions, and varenicline. Based on previous enrollment and retention efforts, it is estimated that n=180 participants will need to enrolled (i.e. sign a consent) in order to reach the n=120 goal, allowing for n=60 participants to be deemed ineligible during screening or to drop out or be removed from the study during the smoking cessation trial.

<u>Method of subject selection</u>

Participants who respond to advertisements (via the internet, local agencies, smoking clinics, medical and dental offices, and in the public transportation system) will undergo a short screening and study information session, with the goal for an intent-to-treat sample of N=120 (n=60/group) who attend the first clinic visit. For eligibility, we will only consider daily smokers with a range of 3-30 cigs/day with drinking levels consistent with the NIAAA hazardous drinking guidelines [>4 drinks in a day for men (>3 for women) and >14 drinks weekly for men (>7 drinks weekly for women)][37,38]. Alcohol and tobacco use disorder diagnoses (DSM-5/ICD-10) will be recorded but will be neither necessary nor sufficient for inclusion. Participants with any major medical or psychiatric contraindications, including severe withdrawal symptoms and history of seizures and DTs when stopping drinking, will be referred to appropriate treatment. Those currently in mild/moderate alcohol withdrawal but without severe complications will be referred to detoxification before being ascertained for study enrollment. We anticipate recruiting 30-35% women and 30-35% racial minorities in the study similar to our prior studies and reflective of the demographics of HDS.

<u>Randomization</u>

A computer number generator will determine randomization into either the Patch+Counseling or VAR+Patch+Counseling groups, stratified by:
   - Sex (Male or Female)

- Smoking (lighter: 3-14 cigs/day; heavier: 15-30 cigs/day)

Inclusion/Exclusion Criteria: These are used for safety and ethical reasons for study medications and to define broad study sample characteristics for an effectiveness-based study:

- Age 18-75 years of age
- Smoke 3-30 cigarettes/day
- Desire to quit smoking as indicated on a smoking stages ladder
- Consume >14 (men) or >7 (women) standard alcohol drinks per week (e.g., 1 drink = 12 oz beer, 5 oz wine, 1.5 oz liquor)
- Not having hepatic panel indices > 2 SD, history of seizures or DTs during alcohol withdrawal, or an unstable medical (e.g., hepatitis, cirrhosis, seizure disorder, recent major cardiovascular event, etc.) or psychiatric disorder (e.g., active hallucinations, severe depression, obsessional thinking, self-injury risking significant blood loss, etc.) deemed by the study physician to be at significant risk for adverse interactions with study medications or measures.
- No history of adverse reactions to varenicline (VAR) or nicotine patch.
- No current suicidal ideation (past 6 months) and no history of major suicide attempts.
- For women of child-bearing potential, not currently pregnant or lactating, and does not have plans to become pregnant in next three months, and able to agree to adequate birth control during study participation.
- Ability to understand, read, and write in English, at least 8th grade education
- Willing and able to sign an informed consent
- Stable residence and contact information.

9. Statistical Analysis

Sample size calculations were derived from data obtained from several studies examining varenicline on smoking and drinking outcomes and our past community-based smoking cessation intervention study. Vareniclines's effect was obtained from prior research on quit rates[50], smoking urge[23], and cognitive functioning[22-23]. For the proposed sample size (N = 60 per group), the power to detect a significant medication effect in the main outcomes will be >= 0.8, for one-sided tests with Type I error of 0.05.

Logistic regression will be used to compare quit rates between groups. In addition, survival analysis will be employed to examine time to relapse and generalized estimation equation (GEE) models with different link functions will be used for longitudinal variables (drinks per week, percent heavy drinking days, neurocognitive functioning, negative affect, tobacco urge, withdrawal), controlling for pre-treatment baseline measures.

10. Potential Risks and Benefits

Potential risks include:

- Possible side effects of varenecline (for those in the augmented treatment condition) which include:
  - *Most Common* - nausea, abnormal (e.g. vivid, unusual, or strange) dreams, constipation, flatulence, and vomiting
  - *Rare* - serious skin reactions, angioedema and hypersensitivity reactions, accidental injury, cardiovascular events, increased effects of alcohol, new or worsening seizures, neuropsychiatric events (i.e. depression, anxiety, suicidal ideation, suicide attempt)

- Possible side effects of Nicotine Patch which include:
  - *Most Common* – Skin irritation (i.e. redness, itching, swelling and rash)

- *Rare*- headaches, dizziness, sleep disturbance, drowsiness, tiredness, difficulty concentrating, nausea, abdominal pain, breathing symptoms, pain in chest or left arm, high blood pressure, and increased heart rate

- Possible side effects of blood draws which include:
  - *Most Common* – discomfort, pain, redness, bruising, and swelling at draw site

- Possible side effects of questionnaires which include:
  - *Rare* – discomfort or mental stress for items assessing current mood or affect (i..e "do you feel down or sad?") and items reporting on substance use and problems

Potential benefits include possible direct benefit from receiving comprehensive smoking cessation treatment at no financial cost. Subjects will also receive medical assessment at no personal expense and will be given appropriate referrals if medical problems are discovered. The health risks of continued smoking and associated factors (i.e., concurrent heavy use of alcohol or recreational drugs, risk for fires or accidents, and secondary harmful effects on their children) will also be reviewed.

11. Monitoring of Safety

During screening, study applicants will undergo a history, complete battery of medical laboratory tests and physical exam to determine their eligibility and safety of their participation in this study. Study applicants will be excluded if they have any major medication or psychiatric conditions that may contraindicate their participation in this study. During the treatment phase of the study, participants will be asked about adverse events at each clinic visit and vital signs will be obtained before administering the study medication. Patients will not receive the medication if they report any signs or symptoms that may contraindicate its administration. Participants will be referred for immediate evaluation by the study physician. Participants will be provided with the contact information for both the study P.I. and physician to report any adverse reactions between study visits.

All unanticipated problems and adverse events (AEs) occurring during the course of the study must be collected, documented, and reported to the Principal Investigator. The occurrence of AEs will be assessed at baseline and each clinic visit during the treatment phase of the study and again during the 26-week follow-up. The PI and study physician will review the AE Forms weekly for events reported as new or continuing. The study investigators will follow all AEs to the point of a satisfactory resolution. A study participant may have their medication discontinued of may be withdrawn from the study if the medically responsible investigator determines it is the best decision in order to protect the safety of a participant. All AEs will be assessed and reported to the IRB (within the specified time or, in the case of internal fatal/life threatening unanticipated events reported immediately), to the FDA, and to the study sponsor via an Investigator Initiated Research Serious Adverse Event Form. The P.I., a licensed clinical psychologist, and study physician will be available to provide necessary referrals for all events of a medical or psychiatric nature.

12. Payment

Participants will not be paid to receive smoking cessation treatment, but will be provided with a small stipend of $60.00 for their time in completing surveys and study measures at the end of treatment (Study Week 12). Only participants completing all measures and surveys through Week 12 will be paid the entire stipend (in the form of a gift card), but pro-rated compensation ($10/visit) will be made available for those who drop out at points prior to the end of treatment. During visits, participants will receive nicotine patches, brief counseling, and study medication (50% of sample) at no charge. Participants will also receive travel reimbursement to and from their study visit in the form of public transit passes or garage parking passes. During the follow-up interview (Week 26) a $40.00 gift card follow-up incentive

will be provided for all participants completing their follow-up within 2 weeks of the initial due date. Those completing after this date will be provided a pro-rated $20.00 gift card.

13. Procedures to Obtain and Record Informed Consent

Subjects will read and sign the written consent form prior to study participation. Subjects will be asked if they have any questions and will be encouraged to exchange their understanding of the study procedures with the researcher. Consent forms will be kept in a separate locked file.

14. Procedures to Maintain Confidentiality

Subjects will be given a four-digit subject code and their name will not appear on any of their data records. This information will be filed in a locked cabinet in the project coordinator's office. Only research personnel will have access to this information.

15. Bibliographic References

1. Dawson, D. A. (2000). Drinking as a risk factor for sustained smoking. Drug and Alcohol Dependence, 59, 235–249.
2. Mokdad AH, Marks JS, Stroup DF, Gerberding JL. Actual causes of death in the United States, 2000. JAMA. 2004;291(10):1238-1245.
3. World Health Organization. 2010. http://www.who.int/nmh/publications/ncd_report_full_en.pdf. Published Last Modified Date|. Accessed Dated Accessed|.
4. DiFranza JR, Guerrera MP. Alcoholism and smoking. J Stud Alcohol. 1990;51(2):130-135.
5. Dani JA, Harris RA. Nicotine addiction and comorbidity with alcohol abuse and mental illness. Nat Neurosci. 2005;8(11):1465-1470.
6. Harrison EL, Desai RA, McKee SA. Nondaily smoking and alcohol use, hazardous drinking, and alcohol diagnoses among young adults: findings from the NESARC. Alcohol Clin Exp Res. 2008;32(12):2081-2087.
7. Hughes JR. Treatment of smoking cessation in smokers with past alcohol/drug problems. J Subst Abuse Treat. 1993;10(2):181-187.
8. Kalman D, Morissette SB, George TP. Co-morbidity of smoking in patients with psychiatric and substance use disorders. Am J Addict. 2005;14(2):106-123.
9. Negri E, La Vecchia C, Franceschi S, Tavani A. Attributable risk for oral cancer in northern Italy. Cancer Epidemiol Biomarkers Prev. 1993;2(3):189-193.
10. Pelucchi C, Gallus S, Garavello W, Bosetti C, La Vecchia C. Cancer risk associated with alcohol and tobacco use: focus on upper aero-digestive tract and liver. Alcohol Res Health. 2006;29(3):193-198.
11. Prabhu A, Obi KO, Rubenstein JH. The synergistic effects of alcohol and tobacco consumption on the risk of esophageal squamous cell carcinoma: a meta-analysis. Am J Gastroenterol. 2014;109(6):822-827.
12. MacKillop J, Kahler CW. Delayed reward discounting predicts treatment response for heavy drinkers receiving smoking cessation treatment. Drug Alcohol Depend. 2009;104(3):197-203.
13. MacKillop J, Amlung MT, Few LR, Ray LA, Sweet LH, Munafo MR. Delayed reward discounting and addictive behavior: a meta-analysis. Psychopharmacology (Berl). 2011;216(3):305-321.
14. Moallem NR, Ray LA. Dimensions of impulsivity among heavy drinkers, smokers, and heavy drinking smokers: singular and combined effects. Addict Behav. 2012;37(7):871-874.
15. Coe JW, Brooks PR, Vetelino MG, Wirtz MC, Arnold EP, Huang J, Sands SB, Davis TI, Lebel LA, Fox CB, Shrikhande A, Heym JH, Schaeffer E, Rollema H, Lu Y, Mansbach

RS, Chambers LK, Rovetti CC, Schulz DW, Tingley FD, 3rd, O'Neill BT. Varenicline: an alpha4beta2 nicotinic receptor partial agonist for smoking cessation. J Med Chem. 2005;48(10):3474-3477.
16. Perkins KA, Mercincavage M, Fonte CA, Lerman C. Varenicline's effects on acute smoking behavior and reward and their association with subsequent abstinence. Psychopharmacology (Berl). 2010;210(1):45-51.
17. Tapper AR, McKinney SL, Nashmi R, Schwarz J, Deshpande P, Labarca C, Whiteaker P, Marks MJ, Collins AC, Lester HA. Nicotine activation of alpha4* receptors: sufficient for reward, tolerance, and sensitization. Science. 2004;306(5698):1029-1032.
18. Jorenby DE, Hays JT, Rigotti NA, Azoulay S, Watsky EJ, Williams KE, Billing CB, Gong J, Reeves KR. Efficacy of varenicline, an alpha4beta2 nicotinic acetylcholine receptor partial agonist, vs placebo or sustained-release bupropion for smoking cessation: a randomized controlled trial. Jama. 2006;296(1):56-63.
19. Koegelenberg CF, Noor F, Bateman ED, van Zyl-Smit RN, Bruning A, O'Brien JA, Smith C, Abdool-Gaffar MS, Emanuel S, Esterhuizen TM, Irusen EM. Efficacy of varenicline combined with nicotine replacement therapy vs varenicline alone for smoking cessation: a randomized clinical trial. Jama. 2014;312(2):155-161.
20. Hays JT, Croghan IT, Schroeder DR, Ebbert JO, Hurt RD. Varenicline for tobacco dependence treatment in recovering alcohol-dependent smokers: an open-label pilot study. J Subst Abuse Treat. 2011;40(1):102-107.
21. Patterson F, Jepson C, Loughead J, Perkins K, Strasser AA, Siegel S, Frey J, Gur R, Lerman C. Working memory deficits predict short-term smoking resumption following brief abstinence. Drug Alcohol Depend. 2010;106(1):61-64.
22. Patterson F, Jepson C, Strasser AA, Loughead J, Perkins KA, Gur RC, Frey JM, Siegel S, Lerman C. Varenicline improves mood and cognition during smoking abstinence. Biol Psychiatry. 2009;65(2):144-149.
23. Austin AJ, Duka T, Rusted J, Jackson A. Effect of varenicline on aspects of inhibitory control in smokers. Psychopharmacology (Berl). 2014;231(18):3771-3785.
24. Childs E, Roche DJ, King AC, de Wit H. Varenicline potentiates alcohol-induced negative subjective responses and offsets impaired eye movements. Alcohol Clin Exp Res. 2012;36(5):906-914.
25. Rollema H, Russ C, Lee TC, Hurst RS, Bertrand D. Functional Interactions of Varenicline and Nicotine With nAChR Subtypes Implicated in Cardiovascular Control. Nicotine Tob Res. 2014.
26. Singh S, Loke YK, Spangler JG, Furberg CD. Risk of serious adverse cardiovascular events associated with varenicline: a systematic review and meta-analysis. Canadian Medical Association journal. 2011;183(12):1359-1366.
27. Ferguson SG, Shiffman S. The relevance and treatment of cue-induced cravings in tobacco dependence. J Subst Abuse Treat. 2009;36(3):235-243.
28. Hitsman B, Hogarth L, Tseng LJ, Teige JC, Shadel WG, DiBenedetti DB, Danto S, Lee TC, Price LH, Niaura R. Dissociable effect of acute varenicline on tonic versus cue-provoked craving in non-treatment-motivated heavy smokers. Drug Alcohol Depend. 2013;130(1-3):135-141.
29. Brandon TH, Drobes DJ, Unrod M, Heckman BW, Oliver JA, Roetzheim RC, Karver SB, Small BJ. Varenicline effects on craving, cue reactivity, and smoking reward. Psychopharmacology (Berl). 2011;218(2):391-403.
30. Ray LA, Lunny K, Bujarski S, Moallem N, Krull JL, Miotto K. The effects of varenicline on stress-induced and cue-induced craving for cigarettes. Drug Alcohol Depend. 2013;131(1-2):136-142.

31. Leeman RF, Huffman CJ, O'Malley SS. Alcohol history and smoking cessation in nicotine replacement therapy, bupropion sustained release and varenicline trials: a review. Alcohol Alcohol. 2007;42(3):196-206.
32. Fridberg DJ, Cao D, Grant JE, King AC. Naltrexone improves quit rates, attenuates smoking urge, and reduces alcohol use in heavy drinking smokers attempting to quit smoking. Alcohol Clin Exp Res. 2014;38(10):2622-2629.
33. Steensland P, Simms JA, Holgate J, Richards JK, Bartlett SE. Varenicline, an alpha4beta2 nicotinic acetylcholine receptor partial agonist, selectively decreases ethanol consumption and seeking. Proc Natl Acad Sci U S A. 2007;104(30):12518-12523.
34. Mitchell JM, Teague CH, Kayser AS, Bartlett SE, Fields HL. Varenicline decreases alcohol consumption in heavy-drinking smokers. Psychopharmacology (Berl). 2012;223(3):299-306.
35. Fucito LM, Toll BA, Wu R, Romano DM, Tek E, O'Malley SS. A preliminary investigation of varenicline for heavy drinking smokers. Psychopharmacology (Berl). 2011;215(4):655-663.
36. McKee SA, Harrison EL, O'Malley SS, Krishnan-Sarin S, Shi J, Tetrault JM, Picciotto MR, Petrakis IL, Estevez N, Balchunas E. Varenicline reduces alcohol self-administration in heavy-drinking smokers. Biol Psychiatry. 2009;66(2):185-190.
37. NIAAA. Helping patients who drink too much: A clinician's guide NIH Publication No. 05–3769. Bethesda, MD: National Institutes of Health. 2005.
38. SAMHSA. National survey on drug use and health. Bethesda, MD: Office of Applied Studies. 2005.
39. Sobell LC, Sobell MB. Alcohol timeline follow-back users' manual. Toronto, Canada: Addiction Research Foundation; 1995.
40. Skinner, HA, Allen, BA. Alcohol dependence syndrome: Measurement and validation. Journal of Abnormal Psychology, 1982; 91(3): 199-209.
41. Heatherton TF, Kozlowski LT, Frecker RC, Fagerstrom KO. The Fagerstrom Test for Nicotine Dependence: a revision of the Fagerstrom Tolerance Questionnaire. British Journal of Addiction. 1991;86(9):1119-1127.
42. Zachary RA, Shipley, W. C. Shipley institute of living scale: Revised manual: WPS, Western Psychological Services 1986.
43. Cox LS, Tiffany ST, Christen AG. Evaluation of the brief questionnaire of smoking urges (QSU-brief) in laboratory and clinical settings. Nicotine and Tobacco Research 2001;3(1):7-16.
44. Bohn MJ, Krahn DD, Staehler BA. Development and initial validation of a measure of drinking urges in abstinent alcoholics. Alcohol Clin Exp Res. 1995;19(3):600-606.
45. Hughes JR, Hatsukami D. Signs and symptoms of tobacco withdrawal. Arch Gen Psychiatry. 1986;43(3):289-294.
46. Sullivan JT, Sykora K, Schneiderman J, Naranjo CA, Sellers EM. Assessment of alcohol withdrawal: the revised clinical institute withdrawal assessment for alcohol scale (CIWA-Ar). Br J Addict. 1989;84(11):1353-1357.
47. Psychological Assessment Resources. Computerised Wisconsin Card Sort Task Version 4 (WCST); 2003.
48. Logan GD. On the ability to inhibit thought and action: A user's guide to the stop signal paradigm. San Diego: Academic Press; 1994
49. Conway AR, Kane MJ, Bunting MF, Hambrick DZ, Wilhelm O, Engle RW. Working memory span tasks: A methodological review and user's guide. Psychon Bull Rev. 2005;12(5):769-786.
50. Hajek P, McRobbie HJ, Myers KE, Stapleton J, Dhanji AR. Use of varenicline for 4 weeks before quitting smoking: decrease in ad lib smoking and increase in smoking cessation rates. Arch Intern Med. 2011;171(8):770-777.

51. Liang K-Y, Zeger, S. L. Longitudinal data analysis using generalized linear models. Biometrika. 1986;73(1):13-22.
52. Asvat Y, Cao D, Africk JJ, Matthews A, King A. Feasibility and effectiveness of a community-based smoking cessation intervention in a racially diverse, urban smoker cohort. Am J Public Health. 2014;104 Suppl 4:S620-627.
53. King A, Sanchez-Johnsen L, Van Orman S, Cao D, Matthews A. A pilot community-based intensive smoking cessation intervention in African Americans: feasibility, acceptability and early outcome indicators. J Natl Med Assoc. 2008;100(2):208-217.
54. Matthews AK, Sanchez-Johnsen L, King A. Development of a culturally targeted smoking cessation intervention for african american smokers. J Community Health. 2009;34(6):480-492.

16. Description of Recruiting Methods

Advertisements will be used to attract heavy drinking smokers for study participation. Advertisements will be placed online (i.e. Craig's List, Marketplace, Chicago Reader Online), in newspapers (i.e. Chicago Red Eye, Chicago Reader, Chicago Tribune), and via flyers posted in research areas (e.g. bulletin boards in Department of Psychiatry), pre-approved areas in the hospital, and pre-approved public areas (e.g. Laundry mats, medical or dental offices, smoking clinics, church bulletin boards, community centers, etc.) with expressed consent from both the IRB and business owner or operator. Patients at the University of Chicago will not be directly recruited through clinician or primary physicians.

Applicants will contact the study to conduct a brief phone interview to assess initial eligibility (i.e. age, smoking status, availability, etc.). Research assistants associated with the study and trained on issues of confidentiality will conduct these phone interviews, scheduling those applicants who are eligible for an in-person screening and providing a list of alternate smoking cessation referrals to those who do not meet basic eligibility criteria.

17. Description of How the Subject's Primary Physician Will be Notified

Patients at the University of Chicago will not be directly recruited through clinician or primary physicians nor will study participation be conducted in concert with any ongoing medical treatment. Information will be conveyed to the study participant directly and s/he will be encouraged to discuss the information with their primary care physician. If the subject prefers and consents, the study physician will discuss pertinent issues with the subject's primary physician as necessary.

18. Description of Anticipated Coordination

The study P.I. will meet with the study physician on a weekly basis to discuss study progress, review adverse events, and to determine course of action on individual cases. A partnership with the Respiratory Health Association (RHA) has been established to rent and utilize space to conduct participant interviews, dispense medications, and provide brief counseling for the effectiveness trial. The P.I. will conduct meetings with RHA on a monthly basis to review study progress and to address scheduling issues as needed. As the RHA has no formal IRB, no formal review or administrative oversight will need to take place with this partnership.

19. Pregnancy Testing

All female candidates who qualify for the study after psychosocial screening will submit to a pregnancy test and must give written assurance of adequate birth control methods during study participation.

20. Rationale for Excluding Women, Minorities, and/or Children from Participation

Children under the age of 18 will not be included in the trial as smoking is illegal in children under 18 and use of nicotine replacement therapy has not been established in children. Women and minorities will be recruited without bias.

II. <u>Summary of Changes to the Protocol</u>

**Protocol changes**	**Approval date**
Added urinary NicAlert test to screening as bioverification of smoker status	October 24, 2017
Obtained approval for addition of placebo pill (identical in appearance to varenicline and provided by Pfizer) to the control (“standard treatment”) arm	November 23, 2017
Updated advertising methods and materials	February 6, 2018
Added collection of urine samples at visits 3 and 4 for potential nicotine and nicotine metabolite assays	March 7, 2018
Updated recruitment methods and materials<br><br>Obtained approval to collect an additional whole blood sample at screening for storage for future genetic analyses	May 24, 2018
Obtained approval for an expanded age range for eligibility from 21-75 years to 21-85 years	January 21, 2020
