# Supplementary material for: Effect of Combination Treatment With Varenicline and Nicotine Patch on Smoking Cessation Among Smokers Who Drink Heavily: A Randomized Clinical Trial
Source: JAMA Netw Open. 2022 Mar 4;5(3):e220951. doi: 10.1001/jamanetworkopen.2022.0951 (PMC8897753; doi:10.1001/jamanetworkopen.2022.0951)
Supplement: Supplement 2. — eMethods. Randomization, Blinding, and Bioverification of Self-reported Smoking Status eReferences [file jamanetwopen-e220951-s002.pdf]

Supplementary Online Content

King A, Vena A, de Wit H, Grant JE, Cao D. Effect of combination treatment with varenicline and nicotine patch on smoking cessation among smokers who drink heavily: a randomized clinical trial. *JAMA Netw Open*. 2022;5(3):e220951. doi:10.1001/jamanetworkopen.2022.0951

eMethods. Randomization, Blinding, and Bioverification of Self-reported Smoking Status

eReferences

This supplementary material has been provided by the authors to give readers additional information about their work.

© 2022 King A et al. *JAMA Network Open*.

eMethods. Randomization, Blinding, and Bioverification of Self-Reported Smoking Status

Randomization and blinding

A 1:1 randomization list was generated by the data manager using a random number generator to assign participants to either varenicline with nicotine patch or placebo with nicotine patch, stratified by sex and smoking level at baseline [light (<10 cigarettes daily) and heavy (≥10 cigarettes daily)]. The data manager, also responsible for preparing the medication for dispensation, was the only unblinded site personnel and otherwise was not involved in the study. All participants, providers, and research staff were blinded to the treatment assignments for the duration of the study.

Bioverification of self-reported smoking status: COVID pandemic effects and determinations

Confirming self-reported smoking status was conducted primarily by an expired air carbon monoxide (CO) breath sample via the Micro+™ Smokerlyzer® (Bedfont Scientific, Ltd, coVita, Santa Barbara, CA). These tests were taken at screening and repeated at each visit during active treatment for all participants. While the majority of study visits were completed prior to the SARS-coV-2 pandemic, for the final thirteen participants (11% of the sample), their final study visit occurred during the initial pandemic outbreak when strict shelter-in-place quarantine restrictions were mandated state-wide for all non-essential workers. We pivoted to alternative methods by telephone, virtual visits or online/mailed surveys to continue to maintain data collection. Nine of these participants reported being smoke-free at their last visit but bioverification was not possible as staff were not able to access equipment as per the mandate to reduce the risk of virus transmission during the early lockdown period.

For biochemical confirmation prior to the pandemic, a priori we chose a CO reading of 10 or less ppm to confirm self-report abstinence, to be consistent with previous large-scale studies [1–4]. In addition, as the sample included a high percentage of lower-income, urban Black smokers in communities with high pollution contaminants[5] and less protection by smoke-free laws[6], we felt this decision was justified. Of note, our criterion considered the combined influence of Black, Biracial, and other minority subgroups on their higher incidence of secondhand exposure [7], cigar and cannabis use[8] , and predominant residence in low-income communities with high concentrations of industry and high levels of both pollution and contaminants[5] . In a re-analysis of the data with a lower CO threshold [6 or less ppm, used in a recent trial of heavy-drinking smokers[9]], the confirmation rate

© 2022 King A et al. *JAMA Network Open*.

remained high (94%). The two participants not confirmed were a Hispanic male and a Black male, each self-disclosing either marijuana smoking, legalized for recreational purposes in Illinois in 2020, or cigar smoking, not included in cigarette smoking abstinence criteria. In these cases, we used a second method, i.e., urine samples assayed by Liquid Chromatography-Tandem Mass Spectrometry (LC-MS/MS; Mayo Clinic Laboratories) that confirmed non-tobacco user (nicotine <10 ng/mL, cotinine 17 ng/mL, nornicotine and anabasine, < 2.0 ng/mL) and use via cigar smoking (nicotine 102 ng/mL, cotinine 1200 ng/mL, nornicotine 17 ng/mL, and anabasine < 2.0 ng/mL). These results departed from a comparison participant who reported continued cigarette use (nicotine and cotinine >1,200ng/mL, nornicotine >120 ng/mL, and anabasine 22 ng/mL). These findings appear to be consistent with the emerging literature on other product use [10–12]. Acosta and colleagues[13] have suggested use of a combination approach, as needed. More stringent CO of 5ppm led to quit rate results in the expected direction, taking into account the two exception cases outlined above, for 37.7% in combined treatment and 24.6% placebo.

© 2022 King A et al. *JAMA Network Open*.

eReferences

1. Hughes JR, Keely JP, Niaura RS, Ossip-Klein DJ, Richmond RL, Swan GE. Measures of abstinence in clinical trials: issues and recommendations. *Nicotine Tob Res*. 2003;5(1):13-25. doi:10.1093/NTR/5.1.13

2. Ebbert JO, Hughes JR, West RJ, et al. Effect of varenicline on smoking cessation through smoking reduction: A randomized clinical trial. *JAMA - J Am Med Assoc*. 2015;313(7):687-694. doi:10.1001/jama.2015.280

3. Gonzales D, Rennard SI, Nides M, et al. Varenicline, an α4β2 nicotinic acetylcholine receptor partial agonist, vs sustained-release bupropion and placebo for smoking cessation: A randomized controlled trial. *J Am Med Assoc*. 2006;296(1):47-55. doi:10.1001/jama.296.1.47

4. Jorenby DE, Hays JT, Rigotti NA, et al. Efficacy of varenicline, an α4β2 nicotinic acetylcholine receptor partial agonist, vs placebo or sustained-release bupropion for smoking cessation: A randomized controlled trial. *JAMA*. 2006;296(1):56-63. doi:10.1001/JAMA.296.1.56

5. *City of Chicago: Air Quality and Health Report*.; 2020. http://www.cityofchicago.org/dam/city/depts/dps/ContractAdministration/StandardFormsAgreements/CDOT_FederallyFunded NonFTA_04202005.pdf.

6. Why tobacco is a racial justice issue. Truth Initiative. https://truthinitiative.org/research-resources/targeted-communities/why-tobacco-racial-justice-issue. Published August 3, 2020. Accessed October 4, 2021.

7. Tsai J, Homa DM, Gentzke AS, et al. Morbidity and Mortality Weekly Report Exposure to Secondhand Smoke Among Nonsmokers-United States, 1988-2014. *Morb Mortal Wkly Rep Expo*. 2018;67(48):1342-1346. https://wwwn.cdc.gov/nchs/data/nhanes/2013-2014/manuals/2013_.

8. Wu LT, Zhu H, Swartz MS. Trends in cannabis use disorders among racial/ethnic population groups in the United States. *Drug Alcohol Depend*. 2016;165:181-190. doi:10.1016/j.drugalcdep.2016.06.002

9. Ray LA, Green R, Enders C, et al. Efficacy of combining varenicline and naltrexone for smoking cessation and drinking reduction: A randomized clinical trial. *Am J Psychiatry*. 2021:appi.ajp.2020.2. doi:10.1176/appi.ajp.2020.20070993

10. Campo L, Polledri E, Bechtold P, et al. Determinants of active and environmental exposure to tobacco smoke and upper reference value of urinary cotinine in not exposed individuals. *Environ Res*. 2016;148:154-163. doi:10.1016/j.envres.2016.03.029

11. Chen J, Kettermann A, Rostron BL, Day HR. Biomarkers of exposure among U.S. cigar smokers: An analysis of 1999-2012 National Health and Nutrition Examination Survey (NHANES) data. *Cancer Epidemiol Biomarkers Prev*. 2014;23(12):2906-2915. doi:10.1158/1055-9965.EPI-14-0849

12. Rodriguez J, Jiang R, Johnson WC, MacKenzie BA, Smith LJ, Barr RG. The association of pipe and cigar use with cotinine levels, lung function, and airflow obstruction: A cross-sectional study. *Ann Intern Med*. 2010;152(4):201-210. doi:10.7326/0003-4819-152-4-201002160-00004

13. Acosta MC, Buchhalter AR, Breland AB, Hamilton DCP, Eissenberg T. Urine cotinine as an index of smoking status in smokers during 96-hr abstinence: Comparison between gas chromatography/mass spectrometry and immunoassay test strips. *Nicotine Tob Res*. 2004;6(4):615-620. doi:10.1080/14622200410001727867

© 2022 King A et al. *JAMA Network Open*.
