# Supplementary material for: Effect of Combination Treatment With Varenicline and Nicotine Patch on Smoking Cessation Among Smokers Who Drink Heavily: A Randomized Clinical Trial
Source: JAMA Netw Open. 2022 Mar 4;5(3):e220951. doi: 10.1001/jamanetworkopen.2022.0951 (PMC8897753; doi:10.1001/jamanetworkopen.2022.0951)
Supplement: Supplement 3. — Data Sharing Statement [file jamanetwopen-e220951-s003.pdf]

Data Sharing Statement

King. Effect of Combination Treatment With Varenicline and Nicotine Patch on Smoking Cessation Among Smokers Who Drink Heavily. *JAMA Netw Open*. Published March 04, 2022. doi:10.1001/jamanetworkopen.2022.0951

Data
Data available: Yes
Data types: Deidentified participant data
How to access data: aking@bsd.uchicago.edu
When available: With publication

Supporting Documents
Document types: Informed consent form
How to access documents: aking@bsd.uchicago.edu
When available: With publication

Additional Information
Who can access the data: researchers whose proposed use of the data has been approved
Types of analyses: for a specified purpose
Mechanisms of data availability: with a signed data access agreement
Any additional restrictions: none
